# Altered resting-state hippocampal functional networks associated with chemotherapy-induced prospective memory impairment in breast cancer survivors

**DOI:** 10.1038/srep45135

**Published:** 2017-03-22

**Authors:** Huaidong Cheng, Wen Li, Liang Gong, Han Xuan, Zhonglian Huang, Hong Zhao, Long Sheng Wang, Kai Wang

**Affiliations:** 1 Department of Oncology, the Second Affiliated Hospital of Anhui Medical University, Hefei, Anhui 230601, China; 2 Department of Neurology, Affiliated Zhongda Hospital, School of Medicine, Southeast University, Nanjing, Jiangsu 210009, China; 3 Collaborative Innovation Centre of Neuropsychiatric Disorders and Mental Health, Neuropsychological Laboratory, Department of Neurology, the First Affiliated Hospital of Anhui Medical University, Hefei, Anhui 230022, China

## Abstract

In this study, we aimed to investigate the intrinsic hippocampal functional connectivity (FC) network and its relationship with prospective memory in patients with breast cancer suffering from chemotherapy-induced cognitive impairment (CICI). Thirty-four breast cancer patients before and after adjuvant chemotherapy (CB and CC, respectively) and 31 age- and education-matched cognitively normal (CN) women were recruited and subjected to a prospective memory task and a resting-state functional magnetic resonance imaging scan. Seed-based functional connectivity analysis was used to compare the hippocampal FC networks between CC and CN groups. Partial correction analysis was used to examine the association between the hippocampal FC network and prospective memory in the CC group. The cancer group that underwent chemotherapy obtained significantly poorer scores than the CN group on mini-mental state examination, verbal fluency test, digit span, and prospective memory examination. Compared to the CN group, CC group showed increased hippocampal connectivity in the frontal and parietal cortex, precuneus, posterior cingulate cortex, and the cerebellum. In addition, the increasing hippocampal FC networks were negatively correlated with prospective memory performance in the CC group. These findings suggest maladaptive hippocampal functioning as a mechanism underlying the impairment of prospective memory in patients experiencing CICI.

Chemotherapy-induced cognitive impairment (CICI) is the damage of cognitive functions in cancer patients during or after chemotherapy that includes impairment in attention, memory, ability to learn or reason, executive function, information processing speed, and visual space perception^1^,^2^,^3^,^4^. Results of a recent meta-analysis suggest that long-term cognitive deficits after chemotherapy in patients with breast cancer are, for the most part, small in magnitude^5^. Our previous study identified a subpopulation of patients with breast cancer who after chemotherapy show prospective memory (PM) impairment^6^. Moreover, it has been indicated that breast cancer survivors exhibit a clear pattern of PM, and that fatigue is a major contributor to this deficit^7^. At present, the neurobiological mechanism underlying prospective memory impairment in breast cancer survivors is still unclear.

Breast cancer survivors frequently report memory problems but about everyday memory functioning in these individuals is not well understood. A crucial aspect of using memory in daily activities is to remember to perform a task at an appropriate time without being explicitly reminded to do so. Prospective memory is a form of memory of a planned action or the intention to perform a task in the future^5^ and is closely associated with daily life. PM has been subdivided into event-based prospective memory (EBPM; remembering to buy food after seeing a food store) and time-based prospective memory (TBPM; remembering to turn on television at 3 pm)^8^. PM failure represents the majority of everyday memory lapses reported by healthy adults^9^. Because PM is such a central aspect of successful daily functioning, understanding the nature, the extent, and the neuropathological mechanisms underlying PM dysfunctionin breast cancer survivors is important.

The neurobiological mechanisms underlying chemobrain are complex and being explored by different disciplines from different perspectives. Neuroimaging is one of the most direct methods to study the mechanism underlying chemobrain^10^. The commonly used imaging methods include functional magnetic resonance imaging (fMRI), positron emission tomography (PET), and event related potential (ERP)^11^. FMRI provides information regarding the function, image, and anatomical factors. Its advantages over PET are high spatial resolution and the fact that it does not involve radiation. FMRI studies suggested that patients with breast cancer subjected to chemotherapy show prefrontal dysfunction. For example, Ferguson and colleagues studied two single twins (one was a patient with breast cancer treated with chemotherapy, the other one had no history of cancer) with fMRI and found that chemotherapy-treated patient showed changes in the frontal lobe function compared to the healthy twin^12^. Resting-state functional magnetic resonance imaging (rs-fMRI) has recently been adopted as a method to explore the mechanisms of breast cancer chemobrain. Bruno and colleagues found that the shortest path was increased and clustering coefficient reduced in the frontal lobe and temporal lobe areas of patients with breast cancer having undergone chemotherapy^13^. This finding promopted an idea that abnormal brain networks in the frontal lobe and temporal lobe brain regions associated with cognitive function may be an important mechanism underlying breast cancer chemobrain. Similarly, Kesler’s study found that breast cancer chemobrain was associated with default mode network (DMN) abnormalities^14^. Patients show damage of DMN after chemotherapy. This could further affect brain functions, such as cognitive ability, by inducing a decline in memory. The hippocampus plays a key role in memory formation, learning^15^, spatial processing^16^, and prospective memory processing^17^. In addition, the association between the amount of chemotherapeutic drugs received and impaired hippocampal neurogenesis in animals and human subjects has been demonstrated^18^. However, the alterations of intrinsic hippocampal functional connectivity (FC) network and their association with the changes in cognitive function related to chemobrain remain to be addressed.

The present study aimed to explore neurobiological mechanisms underlying the prospective memory impairment in 34 breast cancer patients who underwent chemotherapy by using resting-state fMRI to investigate the integrity of hippocampal FC networks. We hypothesized that the intrinsic hippocampal FC network would be impaired in the patient group as compared with the cognitively normal control group, and that the PM performance would be selectively associated with altered hippocampal FC networks in the patient group.

## Results

### Demographic information and neuropsychological data

As illustrated in Table 1, there was no significant difference in the demographic information and GM volume between CC and CN groups (*p* > 0.05). Compared to CN group, CC group showed a significantly deteriorated cognitive function on MMSE, DS, VFT, EBPM, and TBPM (*p* < 0.01).As shown in Table 2, there was no significant difference in the scores of neuropsychological background tests (including MMSE, DS, and VFT), EBPM, and TBPM between CB (breast cancer patients before chemotherapy) and CN groups (*p* > 0.05).As Table 3 shows, the average neuropsychological data scores obtained from the patients having undergone chemotherapy were significantly different from those obtained before chemotherapy (p < 0.01).

### Intrinsic connectivity of the hippocampal networks in CC and CN subjects

**Figure 1:**
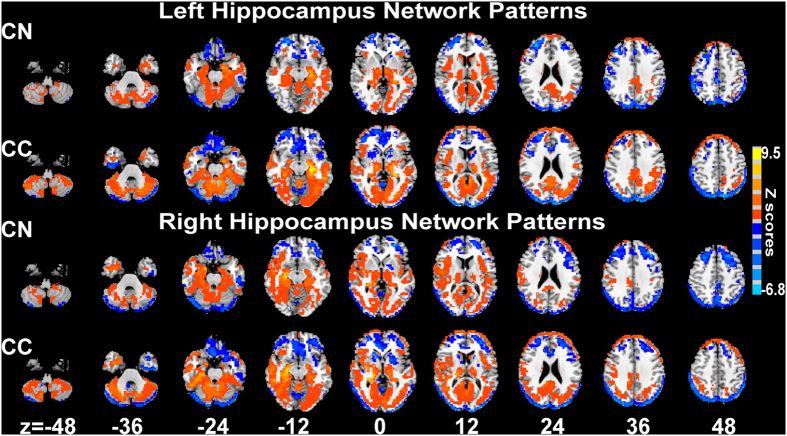
*Resting-state functional connectivity pattern of bilateral hippocampal networks across all participants (p* < 0.05). The results illustrate the different neural constructs of bilateral hippocampal networks for CN and CC subjects revealed by using one sample t-test. Bright color indicates positive connectivity and blue color indicates negative connectivity. Color bar presents Z scores. Abbreviations: CN, cognitively normal controls; CC, cancer patients who underwent chemotherapy.

The patterns of bilateral hippocampal FC networks in both groups are illustrated in Fig. 1. Specifically, for the CN group, the left hippocampus showed a significant positive connectivity with the bilateral insular, thalamus, parahippocampus (PHG), temporal gyrus, lingual gyrus, posterior cingulate cortex (PCC), and the precuneus, while negative connectivity was observed withthe bilateral prefrontal cortex (PFC), orbital frontal cortex (OFC), parietal lobe, and the middle occipital gyrus. Regardingthe right hippocampal networks, the brain regions showing positive connectivity included the bilateral insular, superior and middle temporal gyrus, PCC, thalamus, PHG, fusiform area (FFA), and middle occipital gyrus (MOG), while those showing negative connectivity included the bilateral PFC, OFC, anterior cingulate cortex (ACC), parietal lobe, and the precuneus. In the CC group, the left hippocampal positive connectivity network consisted of the bilateral insular, temporal gyrus, PCC, precuneus, PHG, partly medial prefrontal gyrus, and the inferior parietal lobe (IPL), while the network showing negative connectivity located in bilateral PFC, ACC, OFC, superior parietal lobe, and MOG. The positive right hippocampal network included bilateral insular, PCC, precuneus, PHG, thalamus, posterior temporal gyrus, partly medial prefrontal cortex, and IPL, while the anticorrelated network included the bilateral PFC, OFC, ACC, parietal lobe, occipital gyrus, and partially the anterior part of the temporal gyrus.

### Group-level comparison of functional connectivity within bilateral hippocampal networks

**Figure 2:**
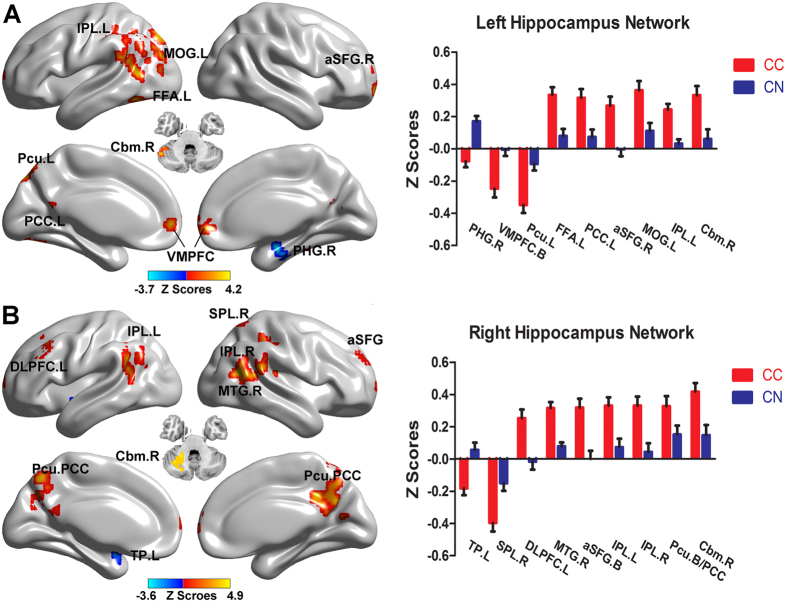
*Brain regions showing different bilateral hippocampal functional connectivity in CC patients compared to CN subjects (p* < 0.01). Brain regions showing significant differencesin bilateral hippocampus functional connectivity networks between the groups are illustrated in (**A**) (left hippocampal network) and B (right hippocampal network). Numerical representation of significant differencesin the bilateral hippocampal networks is described in histograms in A and B. Bright color indicates an increase and blue color indicates a decrease in the connectivity in the CC group as compared with that in the CN group. Color bar presents Z scores. Abbreviations: PHG.R, right parahippocampus; VMPFC. (**B**), bilateral ventromedial prefrontal cortex; Pcu.L, left precuneus; FFA.L, left fusiform area; PCC.L, posterior cingulate cortex; aSFG.R, right anterior part of superior frontal gyrus; MOG.R, right middle occipital gyrus; IPL.L, left inferior parietal lobe; Cbm.R, right cerebellum; TP.L, left temporal pole; SPL.R, right superior parietal lobe; DLPFC.L, left dorsolateral prefrontal cortex; MTG.R, right middle temporal gyrus; IPL.R, right inferior parietal lobe; CC, cancer patients after chemotherapy; CN, cognitively normal control.

We examined the group differences regarding the specificity of bilateral hippocampal network connectivity. As shown in Fig. 2 and Table 4, analysis of the left hippocampal network in the CC group showed increased functional connectivity in several brain regions including the bilateral ventromedial prefrontal cortex (VMPFC), the right anterior portion of the superior frontal gyrus (aSFG) and the cerebellum, as well as in the left precuneus, FFA, PCC, IPL, and MOG, while decreased functional connectivity was observed in the right PHG as compared with that recorded in the CN group. Regarding the right hippocampal network, increased functional connectivity was observed in the bilateral aSFG, PCC, precuneus, and the IPL, right cerebellum, middle temporal gyrus and superior parietal lobe, and left dorsolateral prefrontal cortex (DLPFC), while decreased functional connectivity was present in the left temporal pole (TP).

### Cognitive significance of altered hippocampal functional connectivity networks in CC patients

**Figure 3:**
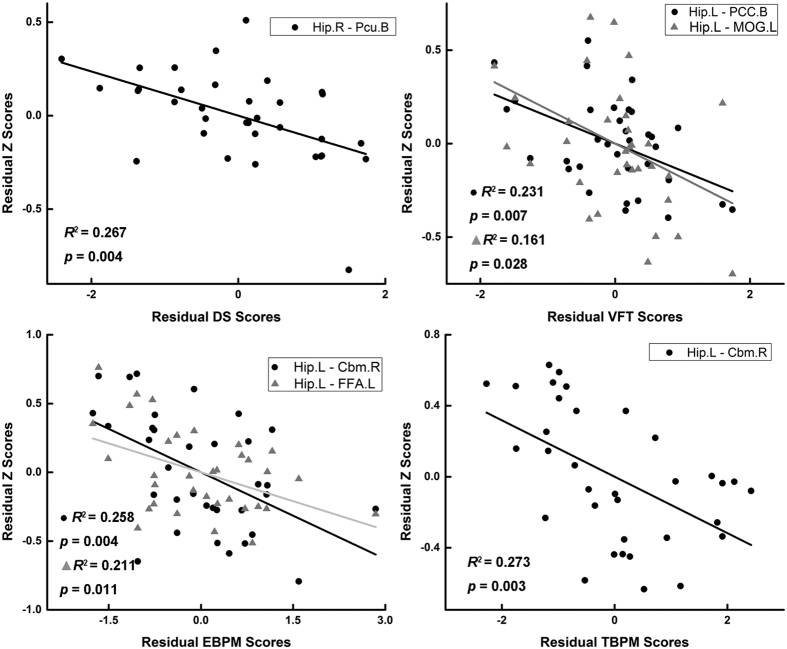
The association between altered hippocampal functional connectivity and behavioral performance in CC patients. Abbreviations: Hip.R, right hippocampus; Hip.L, left hippocampus; DS, digit span; VFT, verbal fluency test; EBPM, event-based prospective memory; TBPM, time-based prospective memory; Pcu.B, bilateral precuneus; PCC.B, bilateral cingulate cortex; MOG.L, left middle occipital gyrus; Cbm.R, right cerebellum; FFA.L, left fusiform area.

The partial correlation analysis revealed that the increase in functional connectivity of hippocampal networks showed a significant negative association with cognitive impairment in CC patients (see Fig. 3). Particularly, the connectivity between the right hippocampus and bilateral precuneus was negatively correlated with digit span performance (*R*^2^ = 0.267, *p* = 0.004), the connectivity between the left hippocampus and bilateral PCC and left MOG was negatively correlated with VFT scores in CC patients (PCC, *R*^2^ = 0.232, *p* = 0.007; MOG, *R*^2^ = 0.161, *p* = 0.028). The left hippocampus and left FFA connectivity were negatively correlated to EBPM scores (*R*^2^ = 0.211, *p* = 0.011). Interestingly, we found that the connectivity between the left hippocampus and the right cerebellum connectivity was negatively correlated to both EBPM and TBPM scores in patient group (EBPM, *R*^2^ = 0.258, *p* = 0.004; TBPM, *R*^2^ = 0.273, *p* = 0.003).

## Discussion

In this study, we investigated if the intrinsic hippocampal functional connectivity was effective in post-chemotherapy breast cancer survivors by using rs-fMRI. We showed that breast cancer survivors after chemotherapy display an extensive cognitive impairment including both EBPM and TBPM. Compared with CN subjects, CC patients showed increased hippocampal FC located mainly in the frontal and parietal cortex, precuneus, PCC, and cerebellum. Importantly, the increase in hippocampal FC networks was negatively associated with prospective memory performance in CC patients.

First, we found that there was no cognitive impairment in CB patients, and that CC patients and CN subjects showed significant differences in cognitive performance including EBPM and TBPM. This indicated that the cognitive impairment in CC patients could be related to chemotherapy. In the present study, there were no significant differences regarding psychological features in breast cancer survivors before and after chemotherapy, and the MMSE, DS, VFT, and PM performance in CC group were all poorer than in CN group, suggesting that the chemotherapy for breast cancer might lead deficits in general cognition and in prospective memory performance. Our finding is in line with most previous studies in that cognitive impairment in chemobrain affects mainly executive functions and presents as a memory deficit^19^,^20^,^21^. However, most studies tested retrospective memory, while the performance of prospective memory was rarely reported^6^,^7^. Paquet and colleagues were the first to report that prospective memory was impaired in breast cancer survivors using Memory for Intention Screening Test, and found that fatigue might modulate this deficit^7^. Our previous study also demonstrated that the EBPM was mainly affected while TBPM was relatively preserved in patients with breast cancer 6.In the present study, the performance in both EBPM and TBPM was found to be impaired in CC patients. The differences in the results between these two studies might arise from different patient traits, as the average age was higher while the educational score was lower in our present study.

According to the Dynamic Multi-process Framework of PM processing^22^,^23^, monitoring and spontaneous retrieval may be used dynamically to support prospective remembering. The monitoring mainly depends on the prefrontal cortex, while the spontaneous retrieval depends on the hippocampal processing^24^. The findings that both EBPM and TBPM are impaired in patients with cancer subjected to chemotherapy and that the monitoring and spontaneous retrieval are inversely correlated to dysfunctional hippocampal connectivity support the common pathway for EBPM and TBPM in spontaneous retrieval processing in PM and further reveal the neurobiological mechanism underlying PM deficits in patients with cancer subjected to chemotherapy.

Second, our findings demonstrated that the intrinsic hippocampal FC network was affected by chemotherapy. The examination of functional connectivity strengths revealed a profile of both decreasing and increasing hippocampal connectivity in CC group compared to CN group. The increasing hippocampal connectivity was mainly found in the frontal and parietal cortex, precuneus, PCC, and the cerebellum, while the hypo- hippocampal connectivity was found in the right parahippocampal gyrus and the left temporal pole. A large number of previous studies have demonstrated that chemotherapy for cancer may influence hippocampal neurogenesis and lead to cognitive impairment and depression^18^,^25^,^26^. Studies using structural MRI also found that the remitted breast cancer patients had a significantly smaller overall gray matter and hippocampal volumes than that of healthy controls and that gray matter atrophy was associated with theabove-mentioned cognitive impairment^27^,^28^,^29^. However, these changes in brain structure appear a long time after the chemotherapy, and the change in hippocampal volume is not consistently reported^18^. Koppelmans *et al*. recruited a relatively large cohort size (184 patients and 368 controls) and the patients have an average 21 years post-treatmentbut did not found significant differences in the hippocampal volume or local gray matter volume between the two groups^30^. According to these data, the structural changes in the brain cannot serve as a prompt and reliable biomarker for early diagnosis of chemotherapy-induced cognitive impairment.

In contrast, the abnormalities in brain function usually appear before the alterations in brain structure and clinical performance^31^. Therefore, detecting alterations in functional connectivity networks might provide anearlier biomarker for CICI diagnosis. Recently, Bruno *et al*. used rs-fMRI and graph analysis and found that the patients with breast cancer treated with chemotherapy displayed alterations in both, the global organization of brain networks and regional network characteristics in the frontal, striatal, and temporal areas^13^, but they did not detect any correlations between networkalterations and cognitive performance in patient group. Kesler *et al*. conducted a multivoxel pattern analysis (MVPA) of DMN functional connectivity and found that the DMN connectivity patterns in chemotherapy group were significantly different compared to non-chemotherapy group and healthy control group. In addition, the DMN classifiers were correlated with memory complaints in chemotherapy group^32^. In the present rs-fMRI study, the patients who underwent chemotherapy mainly showed an increase in hippocampal FC networks compared with CN group, and importantly, such an increase showed a significant negative correlation with working memory, executive function, and prospective memory performance. The inverse relationship between the increase in hippocampal FC and cognitive performance suggests a maladaptive and/or pathogenic mechanism rather than a compensatory phenomenon in CICI. The maladaptive response might reflect an unsuccessful attempt to recruit preserved neuronal areas to compensate for the pathology, as well as a disrupted excitatory-inhibitory balance of damaged networks due to impending pathological processes^33^. Thus, our findings suggest that the alteration of intrinsic hippocampal FC can be considered as a biomarker for early CICI diagnosis.

Third, the present study also found that the connectivity between the left hippocampus and the right cerebellum was negatively correlated to both EBPM and TBPM performance in post-chemotherapy breast cancer patients. Functionally, the hippocampus and the cerebellum are involved in encoding of spatial and temporal information and retrieving of memories^34^,^35^,^36^,^37^. Animal experiments also detected a bidirectional projection between the cerebellum and the hippocampus^38^. More recently, using fMRI, Onuki and colleagues found that the hippocampus and the cerebellum were both activated and that the functional connectivity between the hippocampus and the cerebellum was increased during the temporal and spatial prediction tasks^39^. So far, the cerebellum has rarely been reported as a player in prospective memory processing. Recently, Gonneaud *et al*. found that the cerebellum was more activated during the TBPM task, which might reflect the involvement of time-estimation processes^40^. Halahalli *et al*. reported that the cerebellum was involved in the endogenous-cue prospective memory processing^41^. To our knowledge, the association between altered connectivity between the hippocampus and the cerebellum and the prospective memory has not been reported before. While this phenomenon may be a consequence of chemotherapy-related dysfunction of daily activities in patients with breast cancer, it should be confirmed by further longitudinal studies.

Finally, the present study displays two limitations: first, this was a cross-sectional study and the cohort sizes were relatively small. Further studies using larger cohort sizes are necessary to follow these patients and examine whether the disturbed functional connectivity identified here will change in a long-term after the chemotherapy. Second, the chemotherapy protocols received by different patients were not identical. Different neurotoxic effects of anthracycline versus nonanthracycline-based chemotherapy on cognition in breast cancer survivors have been recently reported^42^. Therefore, the patient group should be subdivided according to different treatments received and these subgroups should be analyzed separately in the future.

In conclusion, this was the first study to investigate intrinsic hippocampal functional connectivity in breast cancer survivors after chemotherapy. Our present findings suggest that both EBPM and TBPM are affected and that the impairment in prospective memory contributes to a disconnection in hippocampal FC networks. These findings propose a functional maladaptive mechanism of prospective memory impairment in the hippocampus of CC patients and indicate that the alterations in the hippocampal FC network might serve as a novel biomarker for CICI in patients with cancer.

## Materials and methods

### Participants

This study included 34 patients with breast cancer before or after adjuvant chemotherapy treatment (CB and CC group) in the Department of Oncology of the Affiliated Second Hospital of Anhui Medical University from June 2013 to June 2015. Thirty-oneage- and education-matched cognitively normal (CN) women were included as controls. All subjects were right-handed with an education of more than five years and were selected according to the following criteria: (1) de novo breast cancer confirmed by postoperative pathology; (2) standard-dose chemotherapy treatment with doxorubicin, paclitaxel, cyclophosphamide, and fluorouracil, but not with hormones; (3) normal cognitive function with the Mini-Mental State Examination (MMSE) score of ≥ 24; (4) normal daily life activities with the KPS score of ≥ 80; and (5) no impairment of vision, hearing, or language. Patients with breast cancer were excluded if the following conditions were present: (1) cachexia and distant metastasis; (2) treatment with hormones; (3) psychiatric symptoms such as anxiety and depression; (4) diseases leading to cognitive dysfunction; (5) a history of alcohol/drug dependence and cognitive therapy; and (6) severe diseases of heart, liver, kidney, brain, and hematopoietic system. The controls were selected according to the following criteria: (1) no complaint of memory loss, no severe diseases, and willingness to undergo examination; (2) aMMSE score of ≥ 24; and (3) normal brain CT and MRI.The study was approved by Research Ethics Committee of the Affiliated Second Hospital of Anhui Medical University, and all subjects providedtheir informed consent.

### Neuropsychological background tests

A battery of tests was administered to all subjects to assess general cognitive and memory functions. MMSE was used to assess the cognitive functions, including time and spatial orientation, short-term memory, calculation, language, and visuospatial skills. The Verbal Fluency Test (VFT), in which the subjects were asked to name as many animals as possible within one minute, was used to evaluate the executive function. The Digit Span (DS) test, in which the subjects were asked to recall a series of numbers after hearing them in a randomized order, was used to measure working memory and attention. The total score was determined by the number of digits recalled in a correct serial order.

### Event-based prospective memory task

Subjects were initially instructed to tap the desk upon detecting two words designating animal species (target event) during the subsequent tasks, and to providetheir telephone number upon completing the tests. Next, subjects were given a word selection task using 30 question cards. On each card, 12 Chinese words were printed. Ten of the 12 words belonged to one category, and the remaining two designated animal species. The experimenter presented each card to the subjects who were instructed to select the card with two words designating animals (target events) and tap the desk. The target events for the prospective memory task were present on the 5^th^, 10^th^, 15^th^, 20^th^, 24^th^, and the 19^th^ card of the word selection task. The subject’s performance on the word selection task was recorded using a method similar to that reported by McDaniel *et al*.^9^ One point was given for each correct response to a target event (total of 6target events). Two points were awarded for remembering to provide their telephone number after the test. Zero points were awarded for an incorrect response to a target event or for forgetting to provide their telephone number. The maximum possible score was 8.

### Time-based prospective memory task

Subjects were instructed to tap the desk at 5-min intervals from the starting time (i.e. at the time points of 5, 10, and 15 min). During the test, subjects were allowed to use a digital clock to check the time. To exclude any visible cues, the clock was located one meter away behind the subject’s right shoulder, so that the subjects had to turn their head to check the time. The clock was set to display 0 hour, 0 min, and 0 s at the beginning of the test. After the clock was started, subjects were administered the number selection task which included 100 cards. On each card, 12 two-digit numbers were printed. Subjects were instructed to select the smallest and the largest numberswithin the cards. The exact time at which the subject responded by tapping the desk was recorded. The number selection task was stopped when the clock indicated 17 min. Two points were given if the subjects responded within the time interval ranging from 10 s before and 10 s after the target time. One point was awarded if the subject responded within the time interval ranging from 30 s before and 30 s after the target time. The maximum possible score for the time-based prospective memory task was 6.

### MRI date acquisition

Imaging was performed using a Siemens Verio 3.0 Tesla scanner (Siemens, Erlangen, Germany) with a homogeneous birdcage head coil at the Affiliated Second Hospital of An Hui Medical University. High resolution spoiled gradient-recalled echo (SPGR) 3D axial images were acquired for anatomical reference. The SPGR parameters were: repetition time (TR) = 1900 ms, echo time (TE) = 2.48 ms, flip angle (FA) = 9°, acquisition matrix = 256 × 256, field of view (FOV) = 240 × 240 mm, thickness = 1.0 mm, gap = 0 mm, number of slices = 176, number of excitations (NEX) = 1.0. Axial resting-state functional connectivity fMRI (R-fMRI) datasets were obtained in 8 min with a gradient-recalled echo-planar imaging (GRE-EPI) pulse sequence. The R-fMRI imaging parameters were: TR = 2000 ms, TE = 25 ms, FA = 90°, acquisition matrix = 64 × 64, FOV = 240 × 240 mm, thickness = 4.0 mm, gap = 0 mm, NEX = 1.0, number of slices = 36. During the data scans, all subjects were instructed to relax and keep their eyes closed, and a pair of stabilizers was used to immobilize their heads.

### Image preprocessing

Pre-processing of the subject data was conducted using a SPM8 toolkit (http://www.fil.ion.ucl.ac.uk/spm)and MATLAB version 7.10 program (The MathWorks, Inc., Natick, MA, USA). The first 10 volumes of the scanning session were discarded to allow for the effects of T1 equilibrations. The remaining 230 volumes were corrected for slice timing, realigned (participants with head motion of more than 3 mm as a maximum displacement in any direction of x, y, and z or 2° of any angular motion were excluded) and then spatially normalized to the standard Montreal Neurological Institute (MNI) EPI template using the default settings and resampling to 3 × 3 × 3 mm^3^ cubic voxels and smoothed with a Gaussian kernel of 6 × 6 × 6 mm. To further reduce the effects of confounding factors, six motion parameters, a global mean signal, the white matter (WM) signal, and the cerebrospinal fluid (CSF) signal were removed from the data using linear regression. No significant difference in head motion was observed among the six groups. Finally, a band-pass filter was applied to retain only low-frequency fluctuations between 0.01–0.08 Hz, and the data was analyzed.

### Voxel-wise based functional connectivity analysis

To avoid misregistration errors, we used an anatomically-based region of interest (ROI). The bilateral hippocampus was selected as ROI using the Slice Viewer in REST software version 1.8 (http://www.restingfmri.sourceforge.net). We intersected bilateral hippocampus in the regions of automated anatomical labeling (AAL) template. For each seed region, a voxel-wise analysis of functional connectivity was performed separately for left and right hippocampus using REST. The mean time series from all voxels within ROI was used as the seed time series. Then the voxel-wise cross-correlation values between the seed region and whole brain voxels were calculated as the strength of functional connectivity. A Fisher’s Z-transformation was applied to improve the normality of the correlation coefficient. Thus, a map of each individual functional connectivity patterns in the hippocampus was obtained.

### Statistical analysis

#### Demographic and neuropsychological data

Two-sample t-test was employed to compare the demographic data and neuropsychological performances among the CC, CB, and CN groups. All values were presented as mean ± standard deviation. Values of *p* < 0.05 were considered as statistically significant. The statistical analyses were performed using the SPSS 20.0 software (SPSS, Inc., Chicago, IL, USA).

#### Gray matter volume correction

To avoid the bias of FC strength due to anatomical variation, the gray matter (GM) volumes were considered as an important covariate in the FC analysis^43^. Optimized voxel-based morphometry (VBM) analysis was performed using the VBM8 toolbox in SPM8 to identify the GM volume in all subjects. The T1-weighted images were segmented to GM, WM, and CSF, and then the segmented GM was normalized and smoothed as the functional image. The final images were regressed out as covariates of FC values to control the influence of GM volume on the strength of FC.

#### Analysis of resting-state functional connectivity

First, asample t-test was used to compare the individual hippocampal patterns in each group, respectively, according to the AlphaSim program based on the Monte Carlo simulation algorithm (*p* < 0.01, cluster sizes > 1080 mm^3^; http://afni.nimh.nih.gov/pub/dist/doc/manual/AlphaSim.pdf). Next, the two-sample t-test was used to compare the differences in the hippocampal connectivity networks in two groups. We removed the covariates such as age, education, and GM volume. Different regions of hippocampal functional connectivity network were visualized with the BrainNet Viewer (http://www.nitrc.org/projects/bnv/). To illustrate the differences numerically in each group and perform further correlation analysis in CC group, the average time series of voxels in these regions were extracted.

#### Partial correlation analysis

In order to explore the association between altered functional connectivity in the hippocampus and cognitive impairment in patients with cancer, we conducted a partial correlation analysis controlling for the covariatessuch as age, education, and GM volume.

## Additional Information

**How to cite this article:** Cheng, H. *et al*. Altered resting-state hippocampal functional networks associated with chemotherapy-induced prospective memory impairment in breast cancer survivors. *Sci. Rep.* **7**, 45135; doi: 10.1038/srep45135 (2017).

**Publisher's note:** Springer Nature remains neutral with regard to jurisdictional claims in published maps and institutional affiliations.

## Figures and Tables

**Table 1:** Demographic information and neuropsychological data frombreast cancer patients after chemotherapy and normal participants.

Parameters	CC(n = 34)		CN(n = 31)		T-value	P-value
	Mean	SD	Mean	SD		
Age(years)	52.00	8.48	50.61	8.32	0.67	0.51
Education(years)	8.12	4.92	8.39	5.16	−0.21	0.83
Chemotherapy cycle	7.47	2.73	—	—	—	—
Gray matter volume	616.65	41.77	621.26	60.11	−0.36	0.72
MMSE	25.59	1.71	29.26	0.93	−10.61	0.00
VFT	6.68	0.84	11.64	2.24	−12.02	0.00
Digit span	5.37	2.24	6.32	0.94	−3.83	0.00
EBPM	1.29	1.08	6.29	1.63	−14.61	0.00
TBPM	3.82	1.36	5.29	0.94	−5.10	0.00

Notes: Data are presented as mean ± SD. Abbreviations: SD, standard deviation; MMSE, mini-mental state examination; VFT, verbal fluency test; EBPM, event-based prospective memory; TBPM, time-based prospective memory; CC, cancer patients after chemotherapy; CN, cognitive normal control.

**Table 2:** Comparison of neuropsychological data from breast cancer patients before chemotherapy versus normal participants

Parameters	CB(n = 34)		CN(n = 31)		T-value	P-value
	Mean	SD	Mean	SD		
MMSE	29.12	0.95	29.26	0.93	−0.60	0.55
VFT	10.85	2.51	11.64	2.24	−1.34	0.19
Digit span	6.38	0.95	6.32	0.94	−0.25	0.80
EBPM	5.71	1.84	6.29	1.63	−1.35	0.18
TBPM	5.09	1.08	5.29	0.94	−0.8	0.43

Notes: Data are presented as mean ± SD. Abbreviations: SD, standard deviation; MMSE, mini-mental state examination; VFT, verbal fluency test; EBPM, event-based prospective memory; TBPM, time-based prospective memory; CB, cancer patients before chemotherapy; CN, cognitive normal control.

**Table 3:** Comparison between neuropsychological data from breast cancer patients before chemotherapy and those collected from patients after chemotherapy.

Parameters	CB(n = 34)		CC(n = 34)		T-value	P-value
	Mean	SD	Mean	SD		
MMSE	29.12	0.95	25.59	1.71	−10.54	0.00
VFT	10.85	2.51	6.68	0.84	−9.19	0.00
Digit span	6.38	0.95	5.37	2.24	−4.16	0.00
EBPM	5.71	1.84	1.29	1.08	−12.06	0.00
TBPM	5.09	1.08	3.82	1.36	−4.24	0.00

Notes: Data are presented as mean ± SD. Abbreviations: SD, standard deviation; MMSE, mini-mental state examination; VFT, verbal fluency test; EBPM, event-based prospective memory; TBPM, time-based prospective memory; CB, cancer patients before chemotherapy; CC, cancer patients after chemotherapy.

**Table 4:** Brain regions showing differences in bilateral hippocampal functional connectivity between the two groups.

Brain region	BA	Cluster size (mm^3^)	MNI Coordinate(RAI)			Peak (*Z* score)
			X	Y	Z	
**Left Hip network**	**CC < CN**					
PHG.R		1404	33	−6	−15	−3.67
**Left Hip network**	**CC > CN**					
VMPFC.B	10	1728	3	54	0	3.24
Pcu.L	7	4104	36	−84	27	3.96
Cbm.R		4860	51	−57	−33	3.68
FFA.L	37	1350	−42	−57	−15	3.85
PCC.L	23	2538	−3	−60	15	4.09
aSFG.R	10	2727	−3	69	6	4.15
MOG.L	31	1836	−27	−81	24	3.27
IPL.L	40	7911	−57	−51	21	3.87
**Right Hip network**	**CC < CN**					
TP.L	48	1998	−48	−6	−3	−3.61
**Right Hip network**	**CC > CN**					
SPL.R	7	1107	6	−66	60	3.09
Cbm.R		3267	33	−60	−30	3.98
MTG.R	21	1782	60	−21	−12	4.76
aSFG.B	10	4644	15	69	3	4.85
IPL.L	39	3510	−42	−60	33	4.00
IPL.R	39	6102	48	−63	18	4.18
Pcu/PCC.B	7/31	11448	−9	−69	33	4.13

Notes:Hip, hippocampus; PHG.R, right parahippocampus; VMPFCB, bilateral ventromedial prefrontal cortex; Pcu.L, left precuneus; FFA.L, left fusiform area; PCC.L, posterior cingulate cortex; aSFG.R, right anterior part of superior frontal gyrus; MOG.R, right middle occipital gyrus; IPL.L, left inferior parietal lobe; Cbm.R, right cerebellum; TP.L, left temporal pole; SPL.R, right superior parietal lobe; DLPFC.L, left dorsolateral prefrontal cortex; MTG.R, right middle temporal gyrus; IPL.R, right inferior parietal lobe; CC, cancer patients after chemotherapy; CN, cognitively normal controls.
